# Entanglement transfer from two-mode continuous variable SU(2) cat states to discrete qubits systems in Jaynes-Cummings Dimers

**DOI:** 10.1038/srep32089

**Published:** 2016-08-24

**Authors:** Du Ran, Chang-Sheng Hu, Zhen-Biao Yang

**Affiliations:** 1 Department of Physics, Fuzhou University, Fuzhou 350002, China

## Abstract

We study the entanglement transfer from a two-mode continuous variable system (initially in the two-mode SU(2) cat states) to a couple of discrete two-state systems (initially in an arbitrary mixed state), by use of the resonant Jaynes-Cummings (JC) interaction. We first quantitatively connect the entanglement transfer to non-Gaussianity of the two-mode SU(2) cat states and find a positive correlation between them. We then investigate the behaviors of the entanglement transfer and find that it is dependent on the initial state of the discrete systems. We also find that the largest possible value of the transferred entanglement exhibits a variety of behaviors for different photon number as well as for the phase angle of the two-mode SU(2) cat states. We finally consider the influences of the noise on the transferred entanglement.

Entanglement is a valuable source in the development of quantum information processing (QIP). In recent years, much attention has been paid to entanglement preparation between two distant qubits, that is realised with, for instance, the optical systems^1^,^2^,^3^, the solid state systems^4^, the ion trap systems^5^,^6^, the cavity quantum electrodynamics (QED)^7^,^8^,^9^,^10^,^11^, the waveguide QED^12^. One widely used method is the entanglement transfer from the continuous variable (CV, including both Gaussian and non-Gaussian) field to the discrete systems^13^,^14^,^15^,^16^,^17^,^18^,^19^,^20^,^21^. More recently, a lot of effort has been devoted to investigating the use of CV systems in QIP. Using CV fields one may carry out quantum teleportation^22^,^23^, quantum computation^24^,^25^ and quantum error correction^26^,^27^,^28^,^29^,^30^. The concepts of quantum cloning^31^,^32^,^33^ and entanglement purification^34^,^35^,^36^ have also been extended to CV systems. Furthermore, tests of quantum nonlocality using CV quantum states have been widely analyzed as well. Efficient resources of entangled CV systems are readily reliable and their field modes can act as reliable information carriers that are of great uses in QIP.

The Gaussian states, due to their well known theoretical structure, can be effectively generated in experiment^37^. These states, such as two-mode squeezed states^14^, twin-beam (TWB) states^19^ have been widely used for entanglement transfer protocols. On the other hand, non-Gaussian states are also important for QIP and may be essential in some cases, e.g., in the case of the distillation of entanglement from two Gaussian entangled states^38^ and of achieving quantum speedup using harmonic oscillators^39^,^40^, that are impossible using only Gaussian operations. There have been protocols showing that non-Gaussian states or non-Gaussian operations are crucial for the realization of entanglement distillation^41^,^42^, quantum error correction^26^, and cluster-state quantum computation^43^,^44^. The uses of non-Gaussian states, such as pair-coherent states (TMC)^19^, entangled coherent states^17^, and photon-subtracted and photon-added two-mode squeezed states^20^, for entanglement transfer are also widely investigated. It has been pointed that for special cases non-Gaussian states (as compared with Gaussian states) or by de-Gaussification process can improve the entanglement transfer process. For instance, it has been shown in ref. 20 that de-Gaussification process by photon-added and photon-subtracted two-mode squeezed vacuum state can improve the entanglement transfer. However, these works only point out that non-Gaussian states can improve entanglement qualitatively.

We investigate here not only qualitatively but also quantitatively the process of entanglement transfer from an anti-correlated two-mode non-Gaussian field to a pair of localized discrete systems via the resonant JC model. The CV systems are initially prepared in a two-mode SU(2) cat state, that has been verified to possess much stronger non-classical properties than that of a single two-mode SU(2) coherent state^45^. Such a state can be used for the test of the fundamental of quantum mechanics, for example, for testing the violations of the Cauchy-Schwarz inequality^45^, and for QIP. We quantitatively connect the entanglement transfer to non-Gaussianity measured based on the Hilbert-Schmidt distance between the state used for the entanglement transfer and a reference Gaussian state^46^ and show that non-Gaussianity has a positive correlation with the transferred entanglement. For both single-photon and multi-photon cases, we find that the adjustable angle *ϕ* of the two-mode SU(2) cat states periodically affects the entanglement transfer process and the largest possible value of the transferred entanglement (*E*_*max*_) changes regularly with the increase of photon number of the two-mode SU(2) cat states. Finally, we find that the transferred entanglement exhibits asymptotic decay under the influences of the main dissipative effects.

The paper is organized as follows: in the next section, we are going to review some properties of the two-mode SU(2) cat states, as well as to introduce the Hamiltonian model for the entanglement transfer. In section III, we consider the entanglement transfer by evaluating the negativity criterion of the reduced atomic density matrix for the entanglement. In IV, we close the paper with some conclusions.

## The two-mode SU(2) cat states and the physical model

We consider the entanglement transfer from a bipartite CV field, the two-mode SU(2) cat states, to a pair of localized discrete systems, by assuming that each CV mode couples to one discrete system via the resonant JC interaction. It can be realized, for instance, by sending each of the two-level atoms through a cavity, for which we will assume that the interaction times and coupling constants are equal for the two subsystems.

The two-mode SU(2) coherent states are defined as^47^,^48^:


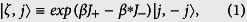


where *β* = *γexp*(−*iφ*) (0 ≤ *γ* ≤ *π*, 0 ≤ *φ* ≤ 2*π*). In terms of Fock states, such states can be expanded as:


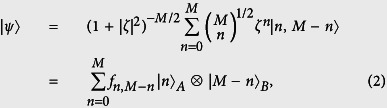


with 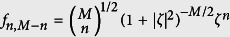, where the subscripts A and B label two different field modes, *M* is the total photon number and *ζ* is a complex parameter related to the partition of photons in the SU(2) coherent states (For mathematical simplicity, hereafter we suppose that *ζ* is real and positive or *ζ* = |*ζ*|).

The two-mode SU(2) cat states are defined as a superposition of two two-mode SU(2) coherent states^45^


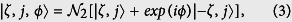


where 0 ≤ *ϕ* ≤ 2*π* is an adjustable angle and 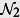 is the normalization factor given by


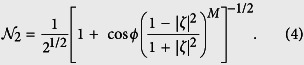


In terms of Fock states, such states can be written as


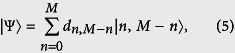


where 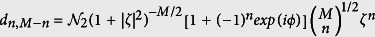.

We use the Von Neumann entropy *S*_*vn*_ of the reduced density matrix of each subsystem to measure the entanglement of a quantum state^49^


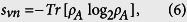


where *ρ*_*A*_ is the reduced density operator when system B is traced out of the whole system, which consists of two subsystems A and B.

For the two-mode SU(2) cat states in Eq. (5), the entropy of the reduced density matrix is defined as


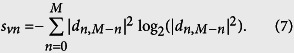


**Figure 1:**
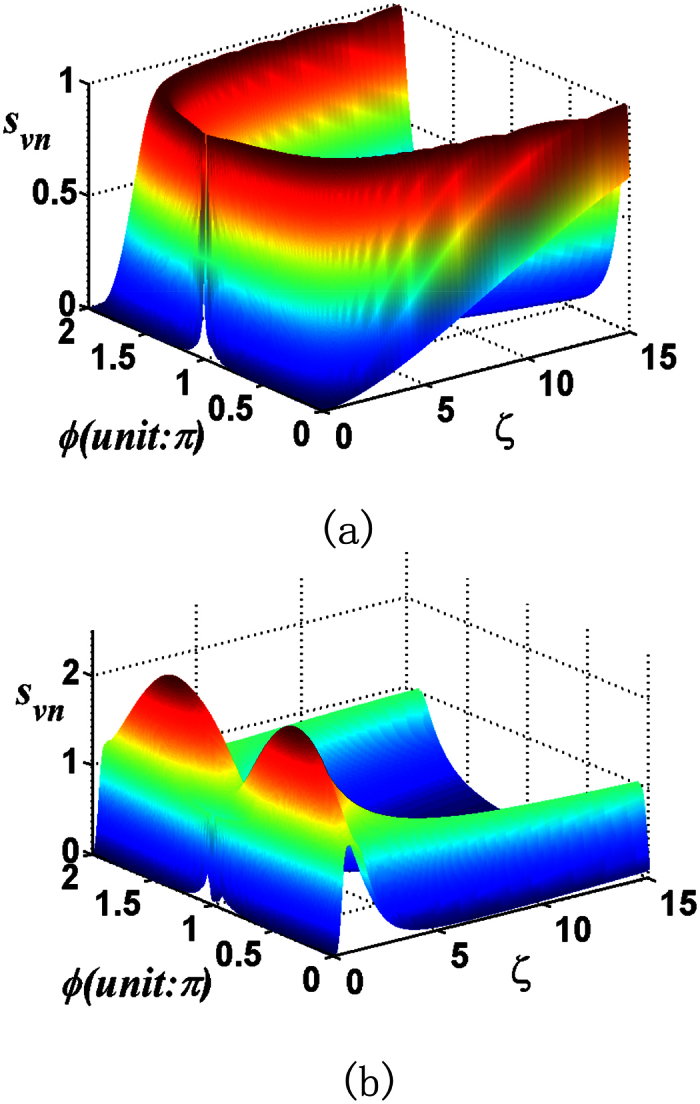
Entropy versus *ζ* and *ϕ* for the two-mode SU(2) cat states. *M* = 1 (**a**), *M* = 5 (**b**).

In Fig. 1, we plot the entropy *s*_*vn*_ versus *ϕ* and *ζ* of the two-mode SU(2) cat states. We can see that there are enormous differences between the single-photon case and the multi-photon case: for *M* = 1, the entropy always possesses maximum value for different *ζ* if we make an appropriate *ϕ*, while for *M* > 1, for example *M* = 5, the entropy only possesses maximum value at *ζ* = 1. And the entropy *s*_*vn*_ seems to be more sensitive to *ϕ* rather than the variation in *ζ* for both two cases. Numerical simulation shows that the entropy increases as the total photon number increases, but the variation trend is gradually decreasing. As the total photon number *M* increases large enough, the growth of entropy will eventually ceases, which is very important for the entanglement transfer that will be shown in the next part.

We then move on to address the interaction of two two-level atoms 1 and 2 with two spatially separate cavities A and B. The interaction Hamiltonian between cavity *j (j* = A, B) and atom *k (k* = 1, 2) in the interaction picture by considering rotating wave approximation is given by (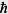 = 1 is assumed)


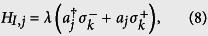


where *λ* is the coupling constant, *a*_*j*_ and 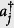 are the bosonic annihilation and creation operators of field mode *j*. The excited and ground states for two atoms are denoted by |*e*〉_*k*_ and |*g*〉_*k*_ (*k* = 1, 2). 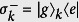 and 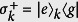 are the lowering and raising operators of atom *k*. Then, the propagation operator of cavity *j* and atom *k* in the basis {|*e*〉_*k*_, |*g*〉_*k*_} is


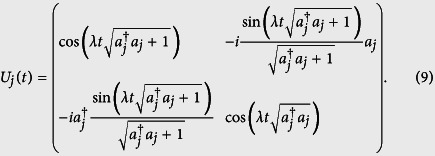


We suppose the atomic system to be initially prepared in


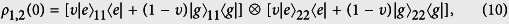


with 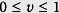.

The evolution by means of the propagation operator *U*(*t*) can be factorized as the product of two resonant JC evolution operators, i.e., *U*(*t*) = *U*_*A*_(*t*) ⊗ *U*_*B*_(*t*). Due to the linearity of the evolution operator, the state of the whole system at time t can be expressed as:


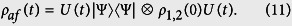


What we are interested in is the entanglement of the subsystem of the atoms, the density matrix of which can be obtained by tracing over the field variable of the density matrix *ρ*_*af*_(*t*) of the whole system, i.e., 
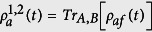
.

## Entanglement Transfer

For the atomic state of Eq. (10) interacting with the two-mode SU(2) cat states, we find the reduced atomic density matrix 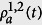 has the form:


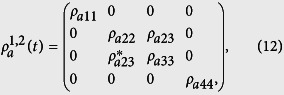


the elements of which are reported in the Appendix.

The presence of the atomic entanglement can be revealed by the Peres-Horodecki criterion^50^,^51^ based on the existence of the negative eigenvalues of the partial transpose of Eq. (12), i.e.,


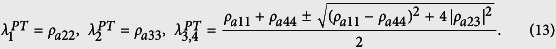


According to the above expressions of the eigenvalues we can see that there is 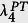 which can be assumed to take negative values. We here adopt the negativity of the partial transpose (NPT) to investigate the entanglement of the two atoms, and, in turn to assess the entanglement transfer. For a two-atom system, the density operator is given by 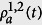, the negativity criterion for the entanglement of the atoms is revealed by the quantity:


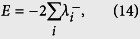


where the sum is taken over the negative eigenvalues 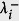 of the partial transpose of the atomic density matrix 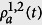. Notice that *E* = 0, 0 < *E* < 1, *E* = 1 denote that the two atoms are separable, partially entangled, and maximally entangled, respectively.

## Entanglement transfer: the single-photon excitation case

The entanglement of the two-mode anti-correlated SU(2) coherent states transferred to a pair of localized discrete systems has been shown somewhere else^52^. Many works have pointed out that non-Gaussian states and de-Gaussifiction states can improve the entanglement transfer from CV systems to discrete systems^19^,^20^, but none of these showed the relationship between the non-Gaussianity and the entanglement transfer quantitatively. This subsection is for a part devoted to this purpose. For the total photon number *M* = 1, the two-mode SU(2) cat states reduce to:


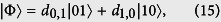


where 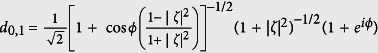 and 
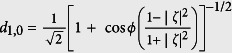

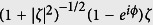
.

Then, we address the relationship between the non-Gaussianity of |Φ〉 and its entanglement transferred to the atomic system quantitatively. Recently, many quantitative measures to assess the non-Gaussianity have been pointed out^46^,^53^,^54^. Here, we adopt the distance between a quantum state *ρ* and a reference Gaussian state *τ*, which itself depends on *ρ*, satisfying the condition as follows: *τ* is the Gaussian state with the same covariance matrix ***σ*** and the same vector ***X*** of the state *ρ*, to measure the non-Gaussianity *δ*[*ρ*] of |Φ〉. The *δ*[*ρ*] is defined as^46^:


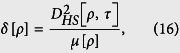


where 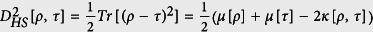 denotes the Hilbert-Schmidt distance between *ρ* and *τ* with *μ*[*ρ*] = *Tr*[*ρ*^2^], *μ*[*τ*] = *Tr*[*τ*^2^] is the purity and *κ*[*ρ*, *τ*] = *Tr*[*ρτ*] denotes the overlap between *ρ* and *τ*. For the state |Φ〉 in Eq. (15), the corresponding reference Gaussian state is *τ*_Φ_ = *R*(*θ*)[*v*(*n*_1_) ⊗ (*n*_2_)]*R*^†^(*θ*), where 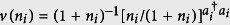 (*i* = 1, 2) is a thermal state with *n*_*i*_ being the average photon number, obtained by mixing two thermal states at a beam splitter of transmissivity cos^2^(*θ*), with 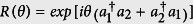. The involved parameter in the reference Gaussian state is an analytical function of the superposition parameter *ζ*.

**Figure 2:**
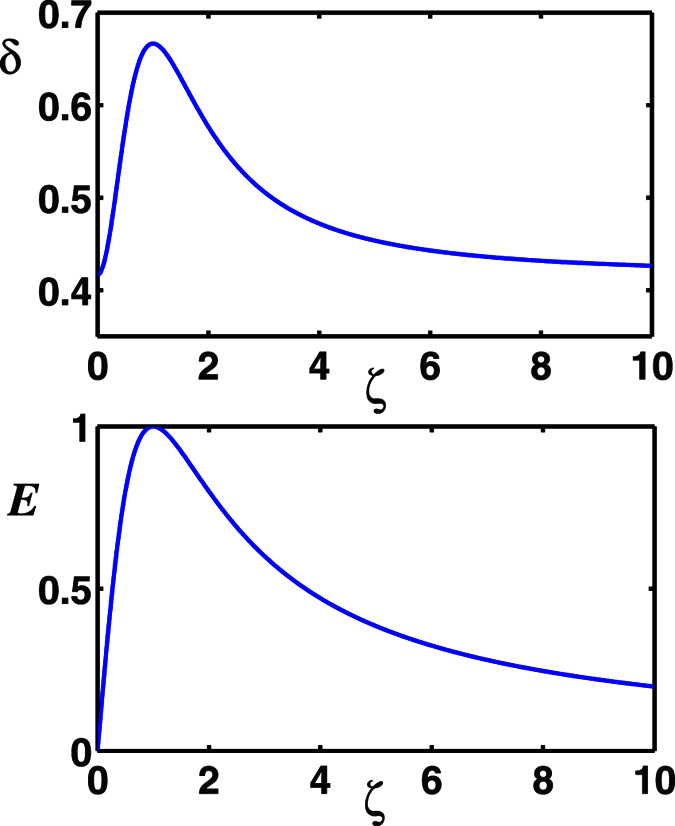
(Top) The non-Gaussianity of |Φ〉 as a function of *ζ*. (Bottom) The entanglement *E* transferred from the two-mode SU(2) cat states with *M* = 1 to two atoms both prepared in the ground state, as a function of *ζ*, with the chosen parameter *λt* = (1/2 + *n*)*π* (n is an integer).

Non-Gaussianity is thus calculated by means of Eq. (16) and is shown in Fig. 2 (Top) as a function of the parameter *ζ*. And the entanglement (quantified by the negativity criterion *E*), transferred from the two-mode SU(2) cat states of *M* = 1 to two atoms that are both in the ground state, versus the parameter *ζ* with the dimensionless parameter *λt* = (1/2 + *n*)*π (n* is an integer) is plotted in Fig. 2 (Bottom). As we can note from Fig. 2 (Top) that the non-Gaussianity of the state |Φ〉 is non-monotonic function of the parameter *ζ*. The non-Gaussianity increases with *ζ* increasing first, and it reaches its maximum at *ζ* = 1 then decreases to *δ*_*min*_ = 0.4167 with *ζ* → ∞. Detailed comparison between the amount of the non-Gaussianity and of the entanglement shows that there exists a positive correlation between the non-Gaussianity and the entanglement. We can quantitatively connect non-Gaussianity to entanglement transfer by *ζ* perfectly, e.g., the maximal entanglement *E*_*max*_ = 1 corresponding to the maximal non-Gaussianity *δ*_*max*_ = 0.6667 both at *ζ* = 1, and *E* = 0.8 corresponding to *δ* = 0.5767 at *ζ* = 0.5 or 2. One may note that for *ζ* = 0, the non-Gaussianity is *δ* = 0.4167, while the entanglement is *E* = 0. But this does not means that for a quantum state with non-Gaussianity not more than 0.4167, the entanglement transfer from this field state to the atomic system can not happen. Obviously, the reason is that for *ζ* = 0, the states |Φ〉 reduce to a product state, which loses entanglement. Also, a lot of works have showed that the Gaussian states can be used for the implementation of entanglement transfer as well. The conclusion of quantitatively connecting non-Gaussianity to entanglement transfer shows the importance of non-Gaussian character of a quantum state to QIP.

What follows is the investigation of entanglement transfer process of the two-mode SU(2) cat states. If we further restrict the parameter to *ϕ* = *π*/2 and *ζ* = 1, such a state becomes a maximally entangled state 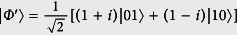. The transferred entanglement from this filed state to the atomic system is similar to the case from the Bell state, which has already been discussed deeply previously^17^.

**Figure 3:**
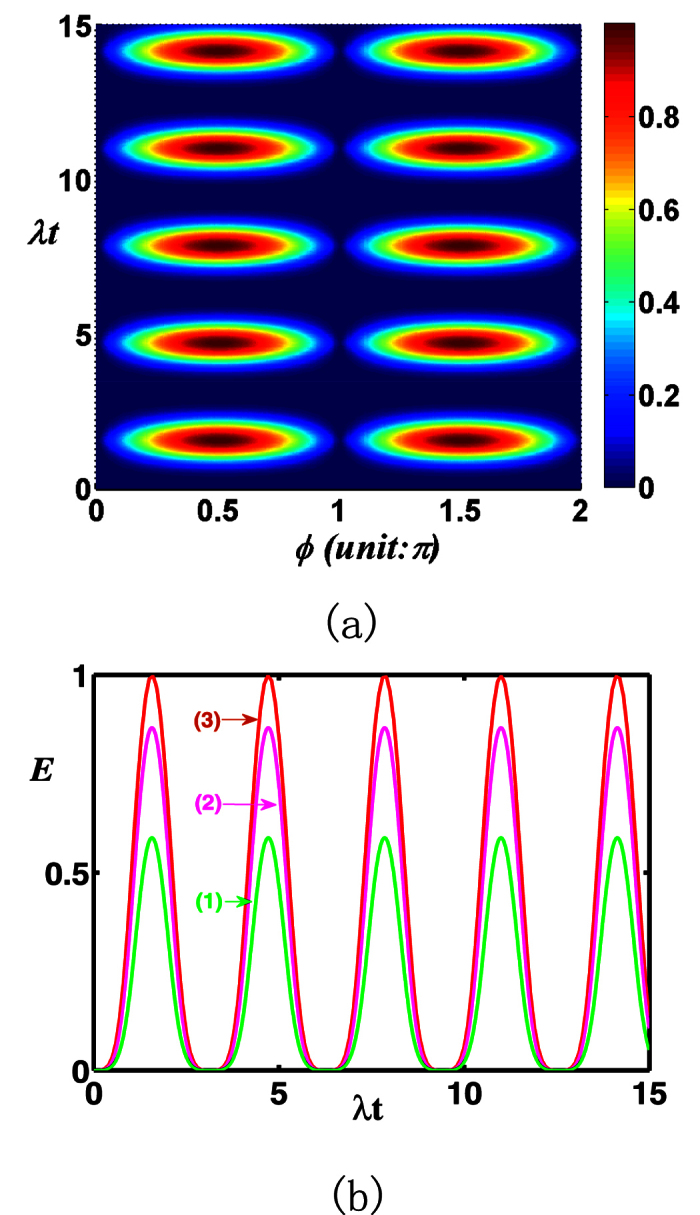
(**a**) For the total photon number *M* = 1 of the two-mode SU(2) cat states, the negativity criterion for the transferred entanglement as a function of *λt* and *ϕ* with the chosen parameters v = 0, *ζ* = 1. (**b**) *ϕ* = *π*/5 (1), *π*/3 (2), *π*/2 (3).

**Figure 4:**
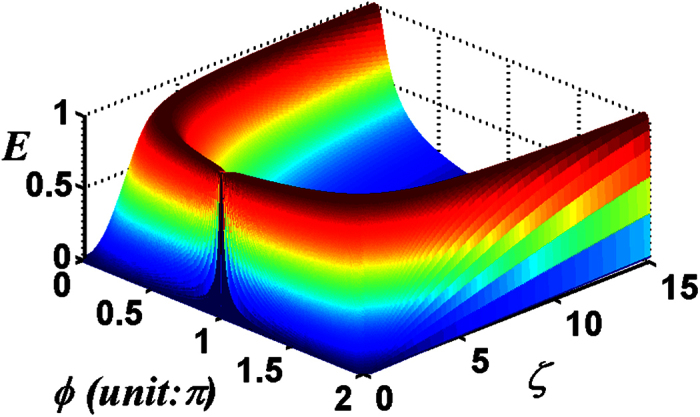
For the total photon number *M* = 1 of the two-mode SU(2) cat states, the negativity criterion for the transferred entanglement as a function of *ζ* and *ϕ* with the chosen parameters *v* = 0, *λt* = 11.

In the following, we will discuss the entanglement transfer under kinds of system parameters. In Fig. 3, we plot the negativity criterion for the transferred entanglement as a function of *λt* and *ϕ* with the chosen parameter *ζ* = 1 and *v* = 0 (both the atoms are initially in the ground state). It can be seen that the negativity *E* is a periodic function of *λt* and *ϕ*, both with a period *π*. It reaches its maximum where the atoms are almost maximally entangled at *λt* = (1/2 + *n*)*π (n* is an integer) and *ϕ* = *mπ*/2 (*m* is an integer). For the case of different initial atomic states, the transferred entanglement is a periodic function of *λt* and *ϕ*, both with a periodic *π* as well, the only difference is that the entanglement at *λt* = (1/2 + *n*)*π* and *ϕ* = *mπ* can not always maintain the same maximum. In Fig. 4, we show the negativity criterion for the transferred entanglement as a function of *ζ* and *ϕ* with the chosen parameter *λt* = 11 (where *E* is independent with *v*^17^) for the initial atomic state |*gg*〉. It is apparent that there is always maximal entanglement for proper *ζ* and *ϕ* (except *ϕ* = *mπ*). After numerical evaluation and comparison, we find that the entanglement (see Fig. 1(a)) of the investigated field state is almost completely transferred to the atomic system at *λt* = 11(~7π/2).

**Figure 5:**
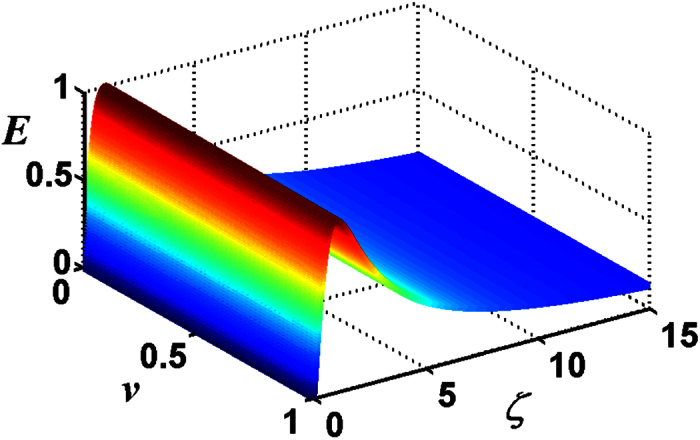
For the total photon number *M* = 1 of the two-mode SU(2) cat states, the negativity criterion for the transferred entanglement as a function of *ζ* and *v* with the chosen parameters *ϕ* = *π*/2, *λt* = 11.

In Fig. 5, we plot the entanglement *E* as a function of the field parameter *ζ* and the initial atomic system parameter *v* with the chosen parameter *ϕ* = *π*/2 and *λt* = 11(~7π/2). For a fixed *ζ*, we can see that the transferred entanglement between the two atoms is independent of the initial atomic state at *λt *~ 7π/2), which in turn proves the entanglement transferred from the two-mode SU(2) cat states for its total photon number *M* = 1 is just similar to the case with Bell states. We have plotted the entropy *S*_*vn*_ versus *ϕ* and *ζ* of the two-mode SU(2) cat state for the total photon number *M* = 1 in Fig. 1. After numerical comparison, we find that the entanglement is completely transferred to the atomic system at *λt *~ 7π/2 regardless of the initial atomic states.

## Entanglement transfer: the multi-photon excitation case

**Figure 6:**
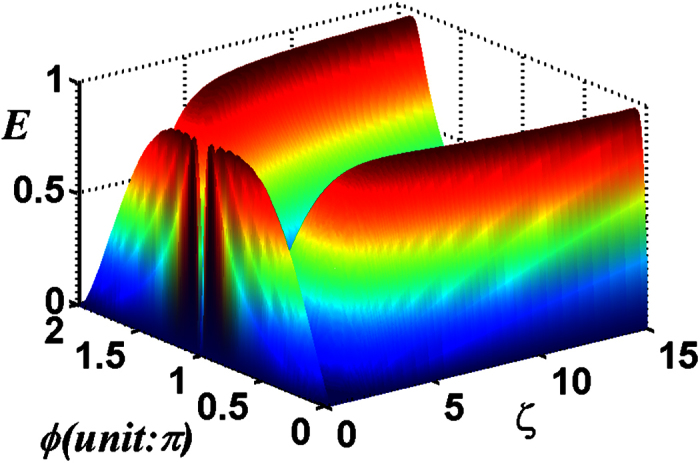
For the total photon number *M* = 5 of the two-mode SU(2) cat states, the negativity criterion for the transferred entanglement as a function of *ζ* and *ϕ* with the chosen parameters *λt* = 7.8, *v* = 0.

We now address the entanglement transfer of the multi-photon excitation. For the total photon number of the two-mode SU(2) cat states, for example *M* = 5, the negativity criterion for the transferred entanglement as a function of *ζ* and *ϕ* with the chosen parameter *λt* = 7.8 and *v* = 0 is shown in Fig. 6. There exhibits an interesting phenomenon in the entropy (as shown in Fig. 1(b)) of the initial field states. The entropy (*s*_*vn*_ < 1) of the initial field states is almost completely transferred to the atomic system, while the entropy (*s*_*vn*_ > 1) transferred to the atomic system decreases with the increase of the entropy. Moreover, the transferred entanglement decreases with the parameter *v* increasing at any *λt*. For the case *v* → 0, the entanglement transfer process will not happen.

**Figure 7:**
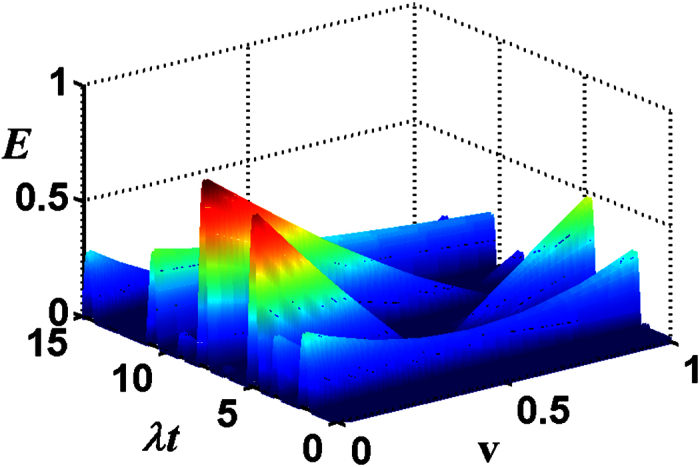
For the total photon number *M* = 5 of the two-mode SU(2) cat states, the negativity criterion for the transferred entanglement as a function of *λt* and *v* with the chosen parameters *ϕ* = *π*/2, *ζ* = 3.3.

Then we discuss the entanglement versus the dimensionless parameter *λt* and the mixedness *v* with the chosen parameter *ϕ* = *π*/2 and *ζ* = 3.3 (where E is the maximum), plotted in Fig. 7. For the multi-photon case, the transferred entanglement is unlike the case with the initial Bell field state, the maximal entanglement is unable to reach 1 ebit and is dependent on *v*, which means that at all times the transferred entanglement is influenced by the mixedness of the initial atomic system.

**Figure 8:**
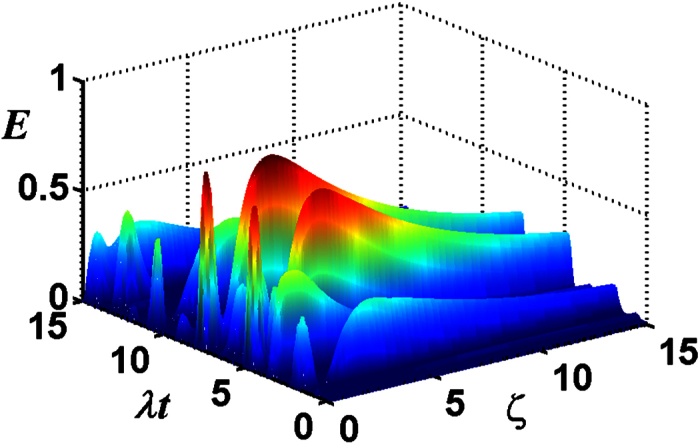
For the total photon number *M* = 5 of the two-mode SU(2) cat states, the negativity criterion for the transferred entanglement as a function of *λt* and *ζ* with the chosen parameters *ϕ* = *π*/2, *v* = 0.

**Figure 9:**
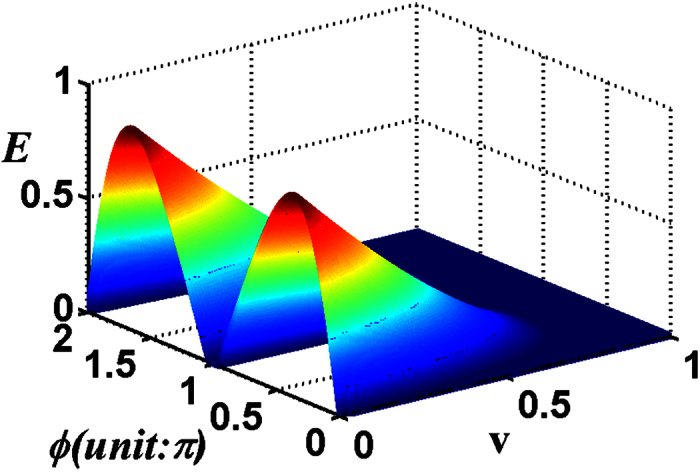
For the total photon number *M* = 5 of the two-mode SU(2) cat states, the negativity criterion for the transferred entanglement as a function of *ϕ* and v with the chosen parameters *ζ* = 3.3, *λt* = 7.8.

The negativity criterion for the transferred entanglement as a function of *λt* and *ζ* with the chosen parameter *ϕ* = *π*/2 for two atoms initially in the ground state is shown in Fig. 8. From Fig. 8, it is apparent that, there are large and well-defined regions for forming the entanglement of two atoms, but the regions are much smaller than that of the single-photon case. Unlike the single-photon case, the negativity E oscillates irregularly as the time evolves. This can be understood as: only one Rabi frequency *λ* is relevant for the single-photon case with initial atomic state |*gg*〉 (for other kinds of initial atomic states it also oscillates irregularly due to more than one Rabi frequency), but the case with more than one Rabi frequency is involved for the multi-photon case. Just similar to the single-photon case, periodic influences of *ϕ* on the multi-photon case is shown in Fig. 9.

In the above, we investigated the process of entanglement transferred from the SU(2) cat states to the discrete atomic system, by taking the total photon number *M* = 5 as an example, aiming to discuss the variance of the transferred entanglement with the system parameters. For both the single-photon case and the multi-photon cases, we find the adjustable angle *ϕ* of the field states periodically affects the entanglement transfer process. The transferred entanglement almost always exists and oscillates (regularly when initial two atoms are both prepared in the ground state for the single-photon case, irregularly for the multi-photon case) as time evolves. The different oscillation behaviour is due to the different number of Rabi frequencies involved (one Rabi frequency *λ* is relevant for the regular oscillation, while multiple Rabi frequencies are involved for the irregular oscillations). The variance of the transferred entanglement with the system parameters for other *M* is similar to the case with *M* = 5.

**Figure 10:**
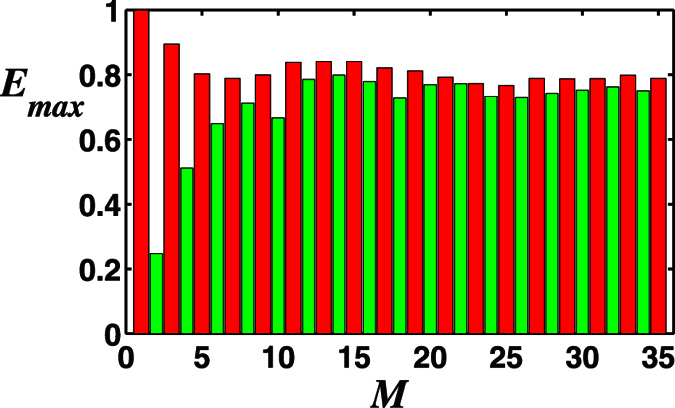
The negativity criterion for the transferred maximum entanglement *E*_*max*_ versus the total photon number *M* of the two-mode SU(2) cat states for the initial atomic state |*gg*〉 with the chosen parameter *ϕ* = *π*/2.

**Figure 11:**
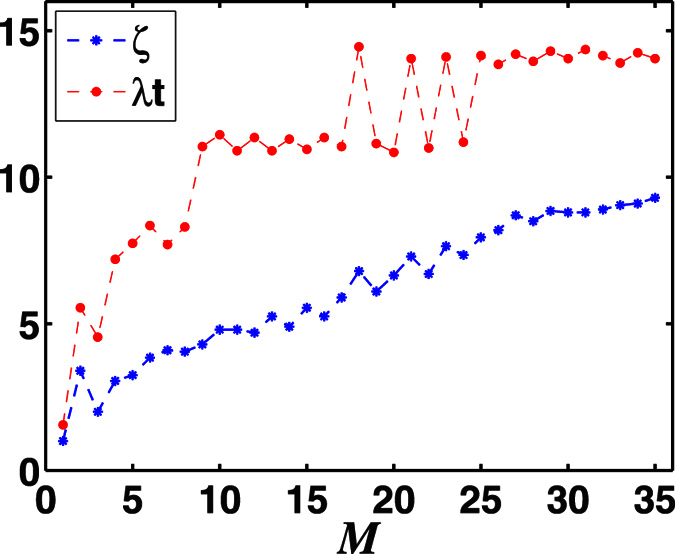
Corresponding to the negativity criterion for the transferred maximum entanglement *E*_*max*_ (Fig. 10), *λt* and *ζ* versus the total photon number *M* of the two-mode SU(2) cat states.

Now we turn to address the relationship between the maximally transferred entanglement *E*_*max*_ and the total photon number *M* of the two-mode SU(2) cat states. In Fig. 10, we plot *E*_*max*_ as a function of *M* with the corresponding *λt* and *ζ* shown in Fig. 11, which shows different influences on the entanglement transfer under even and odd photon number of the two-mode SU(2) cat states. Overall, the largest possible value of the transferred entanglement *E*_*max*_ increases for even photon number and decreases for odd first, then they both remain approximately the same over large *M*. This is due to the reason that the entropy of the two-mode SU(2) cat states is stable when *M* is large enough. And the transferred entanglement *E*_*max*_ for the case with odd photon number is much larger than that for even photon number. In Fig. 11, we can see the evolution time corresponding to the maximal entanglement is slightly similar to that of the single-photon case, mainly focused on (1/2 + *n*)*π* (n is an integer). But as *M* increases, reaching the maximal entanglement needs longer evolution time. While, overall, the field parameter *ζ* corresponding to the maximal entanglement increases with the increase of the photon number *M*.

## The effect of the dissipation and the white noise

In this subsection we take into account the influences of the dissipation and the white noise during the entanglement transfer process. The rates for the atomic spontaneous emission and cavity loss are denoted by *γ*_*a*_ and *κ*_*c*_, respectively. The white noise of atoms and cavities will be characterized in term of the effective photon number *n*_*T*_ and *n*_*C*_^55^,^56^, respectively. The time evolution of the whole atom-cavity system is governed by the master equation in the Lindblad form:


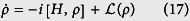


where *H* = *H*_0_ + *H*_*I*,1_ + *H*_*I*,2_, 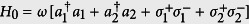, with the Liouvillian given by:


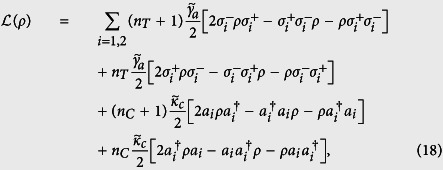


where 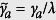 and 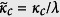 are the dimensionless spontaneous emission and cavity decay rates, respectively.

**Figure 12:**
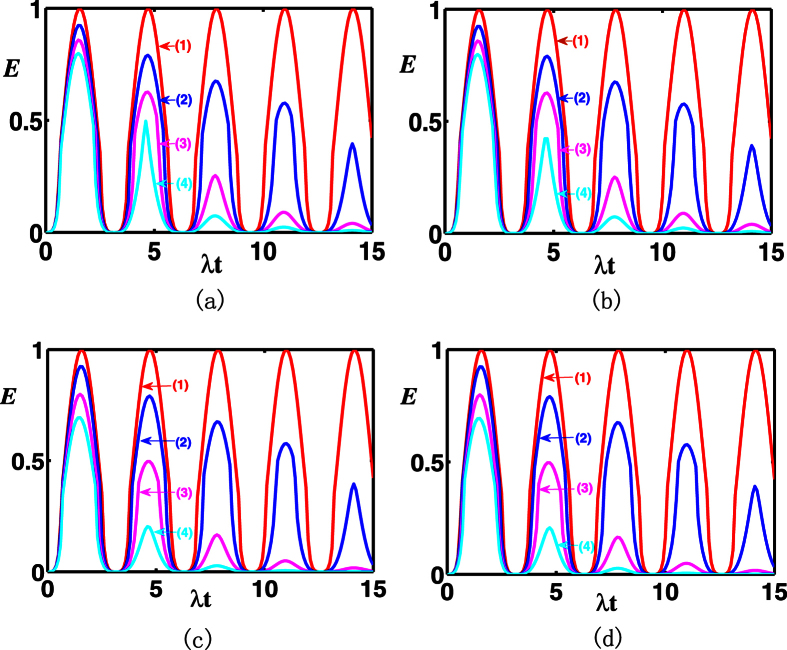
The evolution of transferred entanglement as a function of *λt* for the two-mode SU(2) cat states with *M* = 1, *ζ* = 1, *ϕ* = *π*/2, by assuming two atoms initially in the ground state. (**a**) 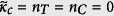, atomic decay rate 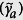: (1) 0, (2) 0.1, (3) 0.2, (4) 0.3. (**b**) 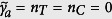, cavity decay rate 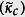: (1) 0, (2) 0.1, (3) 0.2, (4) 0.3. (**c**) 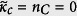, with atomic spontaneous emission and white noise of atoms: (1) 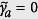, *n*_*T*_ = 0, (2) 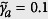, *n*_*T*_ = 0, (3) 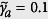, *n*_*T*_ = 2, (4) 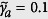, *n*_*T*_ = 4. (**d**) 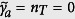, with cavity decay and white noise of cavities: (1) 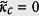, *n*_*C*_ = 0, (2) 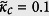, *n*_*C*_ = 0, (3) 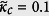, *n*_*C*_ = 2, (4) 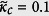, *n*_*C*_ = 4.

In the following, for the case of simplicity, we consider the two-mode SU(2) cat states with *M* = 1, by assuming both the atoms are initially in the ground state. For the chosen parameters, Fig. 12 shows that the entanglement of the two atoms exhibits asymptotic decay, with oscillations of atomic entanglement to finally decay to zero, the larger the damping rate and the white noise are, the smaller the entanglement transfer is. As one may note that the influences of the same atomic decay rate and cavity decay rate are similar. Indeed, there is only slight difference between them, e.g., at *λt* = 14.1, *E* is 0.3953 for 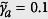, as shown in Fig. 12(a) curve line (2), while, *E* is 0.3918 for 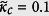, Fig. 12(b) curve line (2). That means the influence of the cavity damping plays a greater role than that of atomic spontaneous emission. One may note the same influence of the white noise of the atoms and cavities from the visual point of view. But the influence of the white noise of the cavities is much greater than that of atoms, e.g., at *λt* = 4.6, *E* is 0.2036 for *n*_*T*_ = 4, as is shown in Fig. 12(c) curve line (4), while *E* is 0.1996 for *n*_*C*_ = 4, as is shown in Fig. 12(d) curve line (4). For the total photon number *M* > 1 of the two-mode SU(2) cat states and different atomic initial states, the transferred entanglement exhibits similar asymptotic decay behavior under the action of a dissipative environment.

## Conclusions

In this paper we address the entanglement transfer from a bipartite CV system to a pair of atomic systems with the assumption that, each CV mode couples to one atom via the resonant JC interaction, and CV systems (two cavity fields) are initially prepared in the two-mode SU(2) cat states. For such a field state, we quantitatively connect its non-Gaussianity to the entanglement transfer and find a positive correlation between them. We then quantify the transferred entanglement by the negativity of partial transpose of the reduced atomic density matrix. We find that the entanglement transferrs from the two-mode SU(2) cat states more efficiently for the single-photon case than for the multi-photon case. For both the single-photon and multi-photon cases, the angle *ϕ* of the field states periodically affects the entanglement transfer process. For the initial atomic state |*gg*〉, we find an interesting phenomenon that the largest possible value of the transferred entanglement *E*_*max*_ increases for even photon number and decreases for odd first, but both remain approximately the same over large *M*. While the corresponding interaction time irregularly increases with the step *π* and the parameter *ζ* gradually increases as *M* increases.

## Additional Information

**How to cite this article**: Ran, D. *et al.* Entanglement transfer from two-mode continuous variable SU(2) cat states to discrete qubits systems in Jaynes-Cummings Dimers. *Sci. Rep.* **6**, 32089; doi: 10.1038/srep32089 (2016).

## Supplementary Material

Supplementary Information
