# Supplementary material for: Entanglement transfer from two-mode continuous variable SU(2) cat states to discrete qubits systems in Jaynes-Cummings Dimers
Source: Sci Rep. 2016 Aug 24;6:32089. doi: 10.1038/srep32089 (PMC4995461; doi:10.1038/srep32089)
Supplement: Supplementary Information [file srep32089-s1.pdf]

SREP-16-14409

The Title: Entanglement transfer from two-mode continuous variable SU(2) cat states to discrete qubits systems in Jaynes-Cummings Dimers

The Authors: Du Ran, Chang-Sheng Hu, and Zhen-Biao Yang

Appendix

The elements of the 4×4 atomic density matrix elements in the standard basis $\{|ee\rangle, |eg\rangle, |ge\rangle, |gg\rangle\}$, by tracing out the field degrees of freedom after the atom-field interaction, are as follows:

$\rho_{a11}(t) = \sum_{i,j=0}^{M} d_{i,j}d^*_{i,j}v^2cos^2(\lambda t\sqrt{i+1})cos^2(\lambda t\sqrt{j+1}) + d_{i,j+1}d^*_{i,j+1}v(1-v)cos^2(\lambda t\sqrt{i+1})sin^2(\lambda t\sqrt{j+1}) + d_{i+1,j}d^*_{i+1,j}v(1-v)sin^2(\lambda t\sqrt{i+1})cos^2(\lambda t\sqrt{j+1}) + d_{i+1,j+1}d^*_{i+1,j+1}(1-v)^2sin^2(\lambda t\sqrt{i+1})sin^2(\lambda t\sqrt{j+1})$,

$\rho_{a22}(t) = \sum_{i,j=0}^{M} d_{i,j-1}d^*_{i,j-1}v^2cos^2(\lambda t\sqrt{i+1})sin^2(\lambda t\sqrt{j}) + d_{i,j}d^*_{i,j}v(1-v)cos^2(\lambda t\sqrt{i+1})cos^2(\lambda t\sqrt{j}) + d_{i+1,j-1}d^*_{i+1,j-1}v(1-v)sin^2(\lambda t\sqrt{i+1})sin^2(\lambda t\sqrt{j}) + d_{i+1,j}d^*_{i+1,j}(1-v)^2sin^2(\lambda t\sqrt{i+1})sin^2(\lambda t\sqrt{j})$,

$\rho_{a33}(t) = \sum_{i,j=0}^{M} d_{i-1,j-1}d^*_{i-1,j-1}v^2sin^2(\lambda t\sqrt{i})cos^2(\lambda t\sqrt{j+1}) + d_{i-1,j+1}d^*_{i-1,j+1}v(1-v)sin^2(\lambda t\sqrt{i})sin^2(\lambda t\sqrt{j+1}) + d_{i,j}d^*_{i,j}v(1-v)cos^2(\lambda t\sqrt{i})cos^2(\lambda t\sqrt{j+1}) + d_{i,j+1}d^*_{i,j+1}(1-v)^2cos^2(\lambda t\sqrt{i})sin^2(\lambda t\sqrt{j+1})$,

$\rho_{a44}(t) = \sum_{i,j=0}^{M} d_{i-1,j-1}d^*_{i,j}v^2sin^2(\lambda t\sqrt{i})sin^2(\lambda t\sqrt{j}) + d_{i-1,j}d^*_{i-1,j}v(1-v)sin^2(\lambda t\sqrt{i})cos^2(\lambda t\sqrt{j}) + d_{i,j-1}d^*_{i,j-1}v(1-v)cos^2(\lambda t\sqrt{i})sin^2(\lambda t\sqrt{j}) + d_{i,j}d^*_{i+,j}(1-v)^2cos^2(\lambda t\sqrt{i})sin^2(\lambda t\sqrt{j})$,

$\rho_{a23}(t) = \sum_{i,j=0}^{M} d_{i,j-1}d^*_{i-1,j}v^2cos(\lambda t\sqrt{i+1})sin(\lambda t\sqrt{i})sin(\lambda t\sqrt{j})cos(\lambda t\sqrt{j+1}) - d_{i,j}d^*_{i-1,j+1}v(1-v)cos(\lambda t\sqrt{i+1}) \times sin(\lambda t\sqrt{i})cos(\lambda t\sqrt{j})sin(\lambda t\sqrt{j+1}) - d_{i+1,j-1}d^*_{i,j}v(1-v)sin(\lambda t\sqrt{i+1})cos(\lambda t\sqrt{i})sin(\lambda t\sqrt{j})cos(\lambda t\sqrt{j+1}) + d_{i+1,j}d^*_{i,j+1}(1-v)^2sin(\lambda t\sqrt{i+1})cos(\lambda t\sqrt{i})cos(\lambda t\sqrt{j})sin(\lambda t\sqrt{j+1})$.
